# Asymmetric Graves’ Orbitopathy

**DOI:** 10.3389/fendo.2020.611845

**Published:** 2020-12-17

**Authors:** Grigorios Panagiotou, Petros Perros

**Affiliations:** ^1^Department of Acute and Intensive Care Medicine, Northwick Park Hospital, London North West University Healthcare NHS Trust, Harrow, United Kingdom; ^2^Department of Endocrinology, Royal Victoria Infirmary, Newcastle-upon-Tyne, United Kingdom

**Keywords:** asymmetry, Graves’ disease, exophthalmos, unilateral, hyperthyroidism

## Abstract

Graves’ Orbitopathy (GO) is an autoimmune orbital disorder usually presenting as a sequala of autoimmune thyroid disease. The presence of GO is associated with increased psychological burden and, in severe cases may cause blindness. While most patients with GO present with bilateral disease, asymmetric or unilateral GO may affect a significant proportion of patients diagnosed with GO. Older age, male sex, active and severe disease correlate with asymmetric disease. However, the exact mechanisms causing asymmetry remain elusive. Herein, we review the literature on asymmetric GO and highlight its differences compared with bilateral GO.

## Introduction

Graves’ orbitopathy (GO) is the most common extrathyroidal feature of Graves’ disease (1) with an estimated prevalence of 10/10,000 persons in European populations (2), while more recent data have shown variable prevalence between 25% and over 50% in Graves’ hyperthyroidism cases (3) worldwide. GO is closely associated with thyroid autoimmunity, and although it is classically linked with hyperthyroidism, GO features have been described in hypothyroid or euthyroid individuals (4).

Autoimmune processes resulting in proliferation of orbital fibroblasts, increased adipogenesis, and extracellular matrix expansion are involved in its pathophysiology (5) and Thyroid Stimulating Hormone (TSH) Receptor Antibodies (TRAb) seem to be a key determinant. In fact, TRAb titer together with smoking, duration of thyroid dysfunction and clinical activity score (CAS) at baseline are regarded as main risk factors for developing GO (6).

The disease most often presents bilaterally and symmetrically with lid retraction, exophthalmos. and diplopia (3) being the most common features. In severe cases GO can be sight-threatening, thus requiring a prompt referral to specialist services (7) upon diagnosis. GO may also cause a psychological and financial burden (8), primarily but not exclusively due to cosmetic concerns, as well as increase the risk for suicide (9), and impairment of quality of life of patients (10).

However, some patients might exhibit asymmetric or unilateral symptoms, for yet unknown reasons. It has been suggested that unilateral GO may progress to bilateral disease (11) and we have also found that asymmetry was associated with more severe and active GO (12). Asymmetry and/or unilateral GO can impose diagnostic challenges (13) and it is therefore important that awareness for asymmetric GO be raised. Clinicians involved in the care of patients with GO need to be able to identify unilateral or asymmetric GO, as timely recognition of asymmetry may expedite referral to specialist services and facilitate further management. However, whether unilateral and/or asymmetric disease represent a distinct variant of GO remains unclear and a better understanding of their underlying mechanisms may provide useful insight on the pathogenesis of GO, in general. Herein, we review the available literature on asymmetric GO and highlight differences compared with bilateral disease.

## Assessment of Asymmetric and/or Unilateral GO

Asymmetry of the human face is well documented (14, 15), while asymmetry in the orbital anatomy of normal human skulls has been shown to be the norm. In a study of 127 human skulls (254 orbits) of individuals aged between newborn to 76 years, asymmetry in orbital anatomy (greater horizontal diameter, greater vertical diameter, orbital perimeter and orbital base area) was present in all cases except four, with variability between 2.47 and 4.47% between the right and left orbit (16). In the same study asymmetry was more prevalent in females and measurements from the right orbit were greater than the left (16). This is of importance, as facial asymmetry in itself can be a cause of significant distress (17).

On the other hand, orbital volume calculation using CT scanning has shown no differences between orbits or gender in a Taiwanese normal population (18). A large study of 653 normal Caucasians subjects aged 21–80 years found asymmetry to be rare (2%), minor (difference in exophthalmos readings <2mm), and unrelated to gender or age. Another study however showed a linear negative correlation between proptosis and age between the ages of 31 and 80 (19), which was also confirmed independently (20). In the latter study, recruiting a large sample (n = 1,063) of normal Iranian subjects including children, a significantly greater proptosis in the right eye compared with the left via exophthalmometry was shown, though the difference was never greater than 2 mm (21).

In the past, asymmetry has been variably defined as difference in proptosis between eyes by ≥2 mm (13), >2 mm (11, 22), or any one of the following criteria: retrobulbar pain or > or =1 grade in soft tissue involvement, and/or of > or =2 mm in exophthalmos, and/or > or =8 degrees in elevation (23), or repeatable asymmetry with regard to more than one symptom and more than one external or anterior segment finding for a duration of two or more visits at least 1 month apart at any time during the initial or follow-up period (23). Radiological criteria for asymmetry, such a right-to-left ratio of more than 1.4 in extraocular muscle diameter, as obtained by CT measurements based on normative data, have also been proposed (24). Unilateral GO is likewise variably defined as one or more features in one eye without any such manifestations in the other eye (20), proptosis >2 mm in one eye with normal examination of the other eye (25), or proptosis difference between eyes >4 mm, and/or if clinical signs and symptoms of GO found unilaterally (26). In our recently published study, asymmetry was defined as bilateral disease with one or more of the following features: difference between the two eyes in exophthalmos by ≥2 mm; difference in palpebral aperture by ≥2 mm; difference in eyelid swelling by ≥1 grades; difference in eyelid erythema by ≥1 grades; difference in conjunctival redness by ≥1 grades; presence of dysthyroid optic neuropathy in one eye only. Unilateral disease was defined as one or more clinical features of GO in one eye without any evidence of GO in the contralateral eye (12). Moreover, disease severity is assessed similarly to bilateral disease, using clinically evaluated standardized tools, such as CAS (27) and Vision, Inflammation, Strabismus, Appearance (VISA) (28). Clearly, there is a need for a consensus in the definitions of asymmetric and unilateral disease.

## Epidemiology of Asymmetric and/or Unilateral GO

Different studies have estimated prevalence of unilateral disease between 4.5 and 14% (13, 25, 29–31), while asymmetry was evident in 9–34% of patients with GO (13, 26, 32). More recently, in our multi-center prospective cohort recruiting 269 newly diagnosed patients from 13 different centers across Europe, we have found a prevalence of 30.9% for asymmetric and 10.7% for unilateral GO (12). However, in a recently published retrospective hospital-based report from India, unilateral disease was much higher at 36% and significantly more common among silent presenters compared to the clinically active group (33). It is important to note that published studies have used different definitions of asymmetry and/or unilateral GO and this might explain the big variation in reported epidemiology among published data. Furthermore, less is known in regards to the epidemiology of unilateral or asymmetric GO in children.

Many risks factors have been associated with asymmetry. Shorter duration of symptoms (29) and thyroid status might be associated with asymmetry. In specific, small studies have suggested that euthyroid and primarily hypothyroid patients develop more asymmetrical GO (34, 35), which tends to present more mildly. In a larger study, Ponto et al. showed that a seven-fold higher risk for unilateral GO in hypothyroid or euthyroid subjects, compared with hyperthyroid GO (26). Moreover, previous studies have shown no difference in regards to race (13) and sex (29), but more recent data, including our recent multicenter study (12) and a cohort of 354 Chinese patients (36), have shown that male sex is associated with asymmetry. In another study, male subjects exhibited asymmetric disease (proptosis and overall asymmetry) three-fold more frequently compared with women, while women with hyperthyroidism demonstrated more symmetry compared with euthyroid and hypothyroid individuals of either sexes (23). With regards to correlation with older age, we have confirmed previous findings supporting higher prevalence and more severe asymmetry in older and especially men (12, 37), in keeping with previously described associations between age and male gender with severity of GO in patients with bilateral disease (38).

## Specific Considerations in Asymmetric GO

It is important that unilateral GO be differentially diagnosed from orbital diseases affecting one eye, such as orbital tumors, such as lymphoma (39, 40). Other differentials affecting the orbit including other orbital tumors or pseudotumors, carotid cavernous fistulae, and dermoid and/or epidermoid cysts need to be excluded, too. Diagnosing unilateral or asymmetric GO without any other signs of GO and especially in the absence of hyperthyroidism or high TRAb titer suggestive of Graves’ disease can be challenging (41) and requires increased clinical awareness and usually imaging of the orbits.

With regards to pathophysiology, the autoimmune processes in asymmetric and unilateral GO, causing expansion of orbital contents seem to be similarly to bilateral disease. However, mechanical, vascular, and inflammatory factors as well as anatomical variances may contribute to development of asymmetric disease.

More specifically, Soroudi et al. have speculated that asymmetric distribution of antigen or inflammatory processes may be the cause of asymmetrical expansion of orbital contents (13), though this was not explored in any studies so far. They have also postulated, that anatomical differences causing differential blood flow or lymphatic drainage may be present (13). Elasticity of orbital septae or other local factors, associated with unilateral triggers such as infections or difference in potential for adipogenesis have also been suggested (12). Others have examined the effect of sleeping position but found no significant correlation with asymmetric GO (32). However, despite previous postulations the exact mechanisms remain elusive. Therefore, more studies are needed to study asymmetric GO and shed light on the mechanisms leading to asymmetry. Such studies may provide further answers in regards to GO development and management.

## Treatment of Unilateral and/or Asymmetric GO

Given that the pathophysiological mechanisms causing unilateral or asymmetric GO appear to be similar to the well-described bilateral GO, treatment of asymmetric GO is generally the same with bilateral disease and is usually dependent on disease severity. However, since asymmetric disease may progress to bilateral GO thereby increasing patients’ anxiety and deteriorating their mental health status and quality of life as alluded to above, it is important that patients are managed promptly. Therefore, the goal in managing asymmetric or unilateral GO should be: a) to ensure symptoms are alleviated and sight is not threatened, similarly to bilateral GO; b) to take measures that may prevent progression to bilateral symptomatology; c) surgical rehabilitation when indicated.

Smoking cessation advice and restoring the euthyroid state are important pillars in the management of asymmetric GO. There is no contraindication in regards to any treatment modality including antithyroid drugs, radioactive iodine, and/or total thyroidectomy for mild asymmetric and/or unilateral GO, while in moderate to severe cases radioiodine should be avoided, similarly to bilateral symmetric disease (42).

In mild cases of asymmetric GO patients may still benefit from selenium supplementation (43). However, there are no prospective data evaluating whether early selenium supplementation in asymmetric GO cases may halt progression to bilateral symptoms. For more severe cases, methylprednisolone infusion (44) and/or administration of immune-modifying therapies, such as rituximab (45) and/or mycophenolate mofetil (46) may need to be considered. Targeted therapies such as teprotumumab, an anti-Insulin Growth Factor 1 (IGF-1) receptor monoclonal antibody, were shown to be effective in patients with active GO (47), but its role in asymmetric or unilateral GO is unexplored. Decompression surgery may be used in appropriate cases.

## Discussion and Conclusions

Asymmetric and unilateral GO are recognized features of GO with variable prevalence among different studies; however, they do not seem to represent a distinct variant of classical GO, rather than the extreme of the spectrum, and thus focused studies are scarce. Therefore, prospective multicenter studies recruiting patients with different socioeconomic, demographic, ethnic, and anthropometric background are needed to better elucidate the epidemiology as well as other parameters of asymmetry and/or unilateral disease. Furthermore, previous studies have shown associations of asymmetric/unilateral GO with disease activity and severity and it is crucial that large cohorts include patients of different clinical status. There is a need for better documentation of the suggestions that asymmetric/unilateral GO might run a milder course or be a prelude to bilateral disease. The mechanisms leading to asymmetric disease discussed are speculative. Animal and mechanistic studies are needed to provide more in depth understanding of the mechanisms leading to disease progression and bilaterality and could identify possible novel therapeutic targets at a molecular level. Wide agreement and consensus among experts on the definition of asymmetry and unilaterality is paramount and will facilitate diagnosis, management, and research into this fascinating clinical entity.

In conclusion, although the available literature is limited, asymmetric and/or unilateral GO tend to be present in older age and male patients and is associated with more active and severe GO. Current evidence suggests that patient presenting with asymmetric or unilateral GO may progress to bilateral disease, which clinicians treating patients with GO need to be aware of. The present mini-review summarizes important information for both clinicians and researchers and also provides the impetus for further research. More specifically, in everyday clinical practice, unilateral disease needs to be differentiated from other pathologies affecting one eye. In the future, mechanistic studies are needed to explore the underlying pathological processes in asymmetric/unilateral GO. Most importantly, longitudinal studies evaluating individuals who are at higher risk for developing bilateral symptoms as well as the effect of various treatments in impeding progression of unilateral and/or asymmetric disease to bilateral GO are warranted.

## Author Contributions

GP: literature search, data acquisition, and writing of the manuscript. PP: conceptualization, literature search, and writing of the manuscript. All authors contributed to the article and approved the submitted version.

## Conflict of Interest

The authors declare that the research was conducted in the absence of any commercial or financial relationships that could be construed as a potential conflict of interest.
